# Simplified single-session EUS-guided transhepatic antegrade stone removal for management of choledocholithiasis in patients with surgically altered anatomy

**DOI:** 10.1093/gastro/goae056

**Published:** 2024-06-25

**Authors:** Tingting Yu, Suning Hou, Haiming Du, Wei Zhang, Jiao Tian, Yankun Hou, Jun Yao, Senlin Hou, Lichao Zhang

**Affiliations:** The Second Hospital of Hebei Medical University, Shijiazhuang, Hebei, P. R. China; The Second Hospital of Hebei Medical University, Shijiazhuang, Hebei, P. R. China; The Second Hospital of Hebei Medical University, Shijiazhuang, Hebei, P. R. China; The Second Hospital of Hebei Medical University, Shijiazhuang, Hebei, P. R. China; The Second Hospital of Hebei Medical University, Shijiazhuang, Hebei, P. R. China; The Second Hospital of Hebei Medical University, Shijiazhuang, Hebei, P. R. China; Jinan University of Second Clinical Medical Sciences, Shenzhen People’s Hospital, Shenzhen, Guangdong, P. R. China; The Second Hospital of Hebei Medical University, Shijiazhuang, Hebei, P. R. China; The Second Hospital of Hebei Medical University, Shijiazhuang, Hebei, P. R. China

**Keywords:** endoscopic ultrasound, transhepatic antegrade stone removal, ERCP, surgically altered anatomy, choledocholithiasis

## Abstract

**Background:**

Endoscopic ultrasound (EUS)-guided transhepatic antegrade stone removal (TASR) has been reserved for choledocholithiasis after failed endoscopic retrograde cholangiopancreatography (ERCP) in recent years. The aim of this study was to evaluate the techniques, feasibility, and safety of simplified single-session EUS-TASR for choledocholithiasis in patients with surgically altered anatomy (SAA).

**Methods:**

A retrospective database of patients with SAA and choledocholithiasis from the Second Hospital of Hebei Medical University (Shijiazhuang, China) between August 2020 and February 2023 was performed. They all underwent single-session EUS-TASR after ERCP failure. Basic characteristics of the patients and details of the procedures were collected. The success rates and adverse events were evaluated and discussed.

**Results:**

During the study period, 13 patients underwent simplified single-session EUS-TASR as a rescue procedure (8 males, median age, 64.0 [IQR, 48.5–69.5] years). SAA consisted of four Whipple procedures, one Billroth II gastrectomy, four gastrectomy with Roux-en-Y anastomoses, and four hepaticojejunostomy with Roux-en-Y anastomoses. The technical success rate was 100% and successful bile duct stone removal was achieved in 12 of the patients (92.3%). Adverse events occurred in two patients (15.4%), while one turned to laparoscopic surgery and the other was managed conservatively.

**Conclusions:**

Simplified single-session EUS-TASR as a rescue procedure after ERCP failure appeared to be effective and safe in the management of choledocholithiasis in patients with SAA. But further evaluation of this technique is still needed, preferably through prospective multicenter trials.

## Introduction

ERCP is considered a standard operation in managing choledocholithiasis [1]. But ∼10%–15% of bile duct stones cannot be managed by using conventional endoscopic stone removal techniques [2], especially in patients with surgically altered anatomy (SAA). For example, some operations, such as gastrectomy with Roux-en-Y reconstruction and hepatoenterostomy, always have a long afferent limb that can make it difficult for a duodenoscope to reach the biliary anastomosis. Thereafter, balloon enteroscopy-assisted ERCP (BE-ERCP) was developed for choledocholithiasis [3]. However, it is still a great challenge, as a balloon-assisted enteroscope may fail to reach and find the papilla or the anastomosis. Even if the balloon enteroscope reaches the biliary opening, cannulation can be very challenging with the enteroscope using limited endoscopic accessories. In addition, stone clearance can be achieved either by pushing them into the duodenum or by percutaneous extraction via percutaneous transhepatic cholangiodrainage (PTCD) [4–6]. Its disadvantages include catheter dislodgement, electrolyte disturbances, the necessity for maintaining access to PTCD for several weeks, causing discomfort, bile leak, and pain.

EUS was originally used as a diagnostic tool. In recent years, it has been used more and more for EUS-guided interventions, such as EUS-guided fine-needle aspiration and EUS-guided biliary drainage (EUS-BD) [7–9]. After achieving EUS-BD, EUS-guided antegrade (EUS-AG) interventions can be performed in biliary benign and malignant diseases. When used for bile duct stones, it is often called EUS-guided transgastrohepatic antegrade stone removal [10] or EUS-guided transhepatic antegrade stone removal (EUS-TASR). Compared with the PTCD approach, EUS-TASR avoids the painful external drains that affect the quality of life for these patients [11].

More and more literature has suggested TASR for bile duct stones in patients with SAA when conventional methods have failed [12–15]. However, the operational process of TASR may need to be simplified and its success rate still needs to be improved. In this manuscript, we reported our experience of simplified single-session TASR for stone removal in patients with SAA.

## Methods

### Study design and subjects

This is a retrospective study on patients with choledocholithiasis undergoing single-session EUS-guided TASR after ERCP failure between August 2020 and February 2023. The procedure was performed by the same associate chief endoscopist at the Second Hospital of Hebei Medical University (Shijiazhuang, China). A portion of participants were excluded, including: (i) participants who were <18 years old; (ii) participants without SAA; (iii) stones with sizes of >15 mm; and (iv) participants with left hepatectomy. Before TASR, all patients underwent attempted ERCP with a gastroscope, colonoscope, or single-balloon enteroscope (Olympus Optical, Tokyo, Japan) with a transparent cap on the tip of the endoscope but failed to reach the papilla.

Case history, relevant laboratory data, endoscopy reports, radiographs, procedural data (puncture point, accessories, length and diameter of stent, procedural findings), clinical success, post-procedural symptoms, and procedure-related adverse events were collected. The trial obtained approval from the Ethics Committee of the Second Hospital of Hebei Medical University. The registration number of the clinical trial is ChiCTR2200066587.

Technical success was defined as successful biliary access followed by cholangiopancreatography, guide-wire placement, and biliary drainage. Clinical success was defined as successful complete stone removal with or without biliary drainage (naso-biliary [NB] tube or plastic stents), including fistula tract dilation and papillary or anastomosis balloon dilation.

### Techniques of simplified single-session EUS-TASR

#### EUS examination

All procedures were performed by using a conventional oblique viewing linear-array scanning echoendoscope (GF-UCT260, Olympus, Tokyo, Japan) connected to an ultrasound processor (EU-ME2 premier Plus, Olympus, Tokyo, Japan).

Standard imaging studies were used to scan the biliary and pancreatic system first. The echoendoscope was placed oriented to the left lobe of the liver and color Doppler imaging was used to differentiate the blood vessels from the bile duct. The bile duct was not selected if it was in close proximity to the periphery of the liver or if a blood vessel existed on the needle passageway to the bile duct. The diameter and distance from the mural wall to the punctured bile duct wall were measured under EUS view to evaluate intrahepatic bile duct (IHBD) access. The number, shape, size, and location of the stones were evaluated in this procedure.

#### EUS-guided antegrade cholangiopancreatography

**Figure 1.**
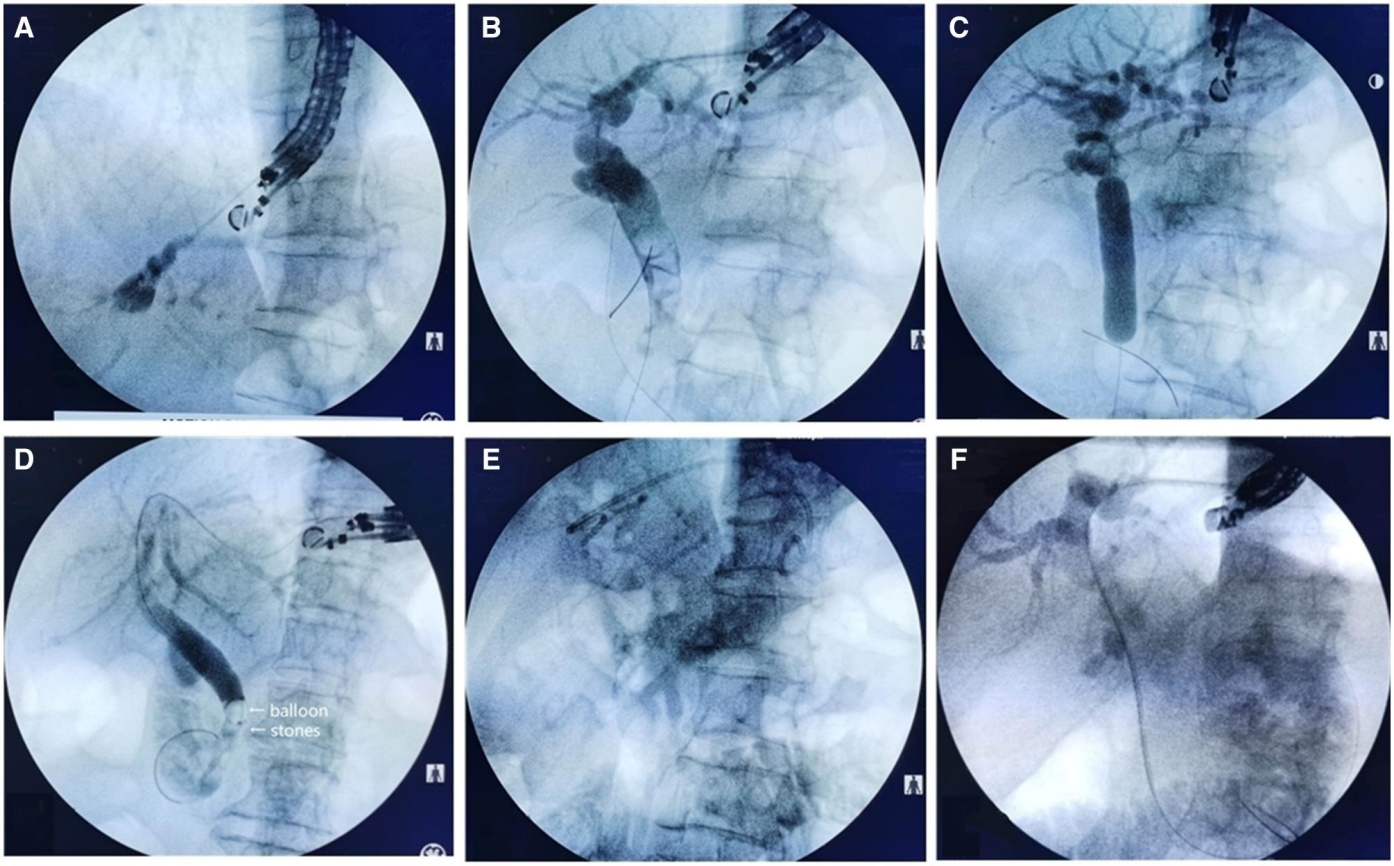
Endoscopic or fluoroscopic images demonstrating the procedural steps. (A) EUS-guided antegrade cholangiopancreatography (EACP) after puncture into the intrahepatic bile duct. (B) Manipulating the guide wire into the common bile duct; a cystotome was used to dilate the fistula. (C) Dilating the biliary opening. (D) Extracting the stone into the duodenum by using a retrieval balloon. (E) Hepaticogastrostomy by using a plastic stent. (F) Naso-biliary tube drainage.

A 19-gauge fine-needle aspiration (FNA) needle (EchoTip, Cook Medical, USA) was used to puncture the selected bile duct. Both fluoroscopy and EUS were used to confirm the access of the needle tip through the digestive wall into the liver parenchyma and then the intrahepatic bile duct. Contrast was then injected for fluoroscopy to evaluate the bile duct (Figure 1A). Normal saline was used to flush the lumen of the needle and a 0.035-inch guide wire with a hydrophilic tip (Jagwire, Boston Scientific, USA) was advanced into the left intrahepatic duct and then into the common hepatic and common bile duct (Figure 1B). We usually advanced the guide wire into the small bowel lumen.

#### Dilation of the hepatoenteric tract and opening of the biliary tree

The FNA needle was withdrawn while the tip of the guide wire was in the intestinal lumen. A 6-Fr cystotome (Endo-Flex, Voerde, Germany) was advanced over the guide wire to dilate the puncture tract. The contrast was then injected again through the dilator catheter or the cystotome to obtain an additional cholangiogram for revaluation of the size and number of stones, and also the anatomy of the biliary system (Figure 1B) [14].

The guide wire was then advanced downstream through the papilla or the anastomosis until it coiled in the small bowel lumen. Coordinated movements of the guide wire and the catheter had to be used at this stage and sometimes even a triple lumen sphincterotome (TRI-25M, Wilson-Cook Medical Inc., Ireland) was be applied to change the direction of the guide wire.

Under fluoroscopy, a dilatation balloon (QBD, Wilson-Cook Medical Inc., Ireland) was passed over the guide wire to the papilla or the hepatoenteric anastomosis to dilate the biliary opening (Figure 1C).

#### Stone removal and stent or NB tube placement

A retrieval balloon (TRI-EX, Wilson-Cook Medical Inc., Ireland) was then advanced proximal to the most distal stone. The balloon was inflated and used to push the stone into the small bowel through the dilated biliary opening (Figure 1D). After complete stone clearance, we injected contrast through the balloon to obtain a clean cholangiogram that was free of choledocholithiasis. An 8-Fr double-pigtail plastic stent (Zimmon, Wilson-Cook Medical Inc., Ireland) (Figure 1E) or a NB tube (7-Fr, Cook Ireland Ltd, Ireland) (Figure 1F) was placed after stone removal for all patients to avoid possible bile leak and hemorrhage. This procedure can also be called EUS-guided hepaticogastrostomy (EUS-HGS) or EUS-guided hepaticojejunostomy (EUS-HJS). After confirmation of the absence of residual stones in the bile duct by using computed tomography (CT) or cholangiography, the stent was removed 1 month later using a duodenoscope (Olympus) and the NB tube was removed 2 weeks later (Supplementary Table 1).

## Results

### Baseline characteristics

During the study period, a total of 13 patients (8 males, median age, 64.0 [IQR, 48.5–69.5] years) with SAA underwent rescue simplified single-session TASR. The types of SAA included one Billroth II gastrectomy, four Whipple procedures, four gastrectomy with Roux-en-Y anastomoses, and four hepaticojejunostomy with Roux-en-Y anastomoses. Five cases with native papilla accounted for 38.5% of the total. The median length of hospital stay and postoperative hospital stay were 9.0 (IQR, 8.0–16.0) and 5.0 (IQR, 3.0–6.0) days, respectively (Table 1).

**Table 1.** Clinical and procedural characteristics

Characteristic	TASR (*n *=* *13)
Age (years) (IQR)	64.0 (48.5, 69.5)
Sex, *n* (%)	
Male	8 (61.5)
Female	5 (38.5)
Fever, *n* (%)	8 (61.5)
Native papilla, *n* (%)	5 (38.5)
Roux-en-Y anatomy, *n* (%)	8 (61.5)
Type of SAA, *n* (%)	
Billroth II gastrectomy	1 (7.7)
Whipple	4 (30.8)
Gastrectomy with Roux-en-Y anastomosis	4 (30.8)
Hepaticojejunostomy with Roux-en-Y anastomosis	4 (30.8)
Bile duct width (mm) (IQR)	5.0 (3.8, 5.7)
Distance between bile duct and puncture site (mm) (IQR)	30.1 (23.5, 37.7)
Operation time (min) (IQR)	60.0 (57.5, 70.0)
Number of stones, *n* (%)	
≤3	10 (76.9)
>3	3 (23.1)
Size of stone, *n* (%)^a^	
≤10 mm	4 (30.8)
>10 mm	9 (69.2)
Length of hospital stay (days) (IQR)	9.0 (8.0, 16.0)
Postoperative hospital stay (days) (IQR)	5.0 (3.0, 6.0)
Adverse events, *n* (%)	2 (15.4)

a The size of the stone refers to the diameter of the largest stone.

TASR = endoscopic ultrasound-guided transhepatic antegrade stone removal, SAA = surgically altered anatomy.

### Simplified EUS-TASR procedure

Standard EUS examination in every patient allowed clear visualization of the IHBD in the left lobe. The median procedure time was 60.0 (IQR, 57.5–70.0) minutes. In all patients, the bile duct of segment II was chosen. The median diameter of the punctured bile duct was 5.0 (IQR, 3.8–5.7) mm on EUS. The median distance between hepatic duct and puncture site was 30.1 (IQR, 23.5–37.7) mm. The IHBD was successfully punctured in all of the 13 patients (100%) (Table 1).

Plastic stents were used in 11 of the patients (84.6%) and an NB tube in Case 1 (7.7%). In Case 4, a stent or NB tube was not placed due to the guide wire’s having dropped out from the biliary system after stone extraction. Case 8 turned to laparoscopic bilioenteric anastomosis 3 days later because of bile leakage and acute peritonitis. For this patient, the stent was removed during the operation. In Case 1, the NB tube was removed after 15 days. The other patients’ stents were removed 1 month later.

The technical success rate was 100%, but the overall clinical success rate was 92.3% (12/13). In one patient (Case 8), the guide wire could not pass the anastomosis, so we only placed a stent between the gastric wall and the common hepatic duct.

### Adverse events

Procedural-related adverse events developed in Cases 4 and 8 (15.4%). Asymptomatic abdominal fluid collection was found in Case 4, who did not have a stent or NB tube to drain the bile. Two days after TASR, CT found fluid between the stomach and the spleen. Another 10 days later, a CT scan showed that the fluid had been absorbed. Bile leak and bile peritonitis occurred in Case 8 and he underwent laparoscopic surgery later. None of the patients had post-procedure hemorrhage and pancreatitis.

## Discussion

Endoscopic transpapillary therapy has been used as the gold-standard therapy for the removal of common bile duct stones [16] but SAA often leads to therapeutic ERCP failure because of the inaccessible biliary tree [17]. For this group of patients, the PTCD method can also be a choice but it is associated with a decrease in patient quality of life, as it can cause cosmetic issues and pain at the puncture site [18]. BE-ERCP is also being used increasingly for benign biliary diseases in patients with SAA and has been shown to be effective and safe, although it is not always successful. The technical success rate for biliary cannulation is 62.5%–99.2% using forward-viewing scopes including gastroscopes and enteroscopes in a Billroth II reconstruction [19] and BE-ERCP has a procedural success rate of only 61.7% in patients with Roux-en-Y reconstruction [20].

EUS-guided antegrade treatment by using a one-stage or two-stage procedure for benign biliary diseases in patients with SAA has been developed for ERCP failure cases in recent years [7]. However, the two-stage procedure is complex and the success rate for one-stage TASR is low [14, 15]. The technical success rate and clinical success rate were as high as 100% and 92.3%, respectively, in our evaluation of simplified single-session TASR and the incidence of adverse events was lower (15.4%) than that for ERCP (25.6%) [21]. We consider that the key to improving the technical success of simplified single-session TASR depends on a simplified process and the application of a 0.035-inch guide wire and co-axial cystotome.

Successful puncture was achieved in all of the patients (100%). A larger bile duct can make puncture easier. The literature also shows that a bile duct diameter of >5 mm and a distance from the gastric mural wall to the punctured bile duct wall of 1–3 cm may be suitable for successful puncture [22].

All of our patients had their bile ducts accessed through liver segment II. Compared with liver segment III, segment II has a relatively straight course toward the downstream of the biliary system, which can make the sequential antegrade interventions easier to perform. However, accessing segment II is not always possible because of blood vessels on a puncture line and the risk of trans-esophageal puncture [14]. Therefore, the puncture point and puncture path were evaluated once and then once again carefully.

Although a 22-gauge FNA needle and a 0.018-inch guide wire could also be used if the targeted bile duct is not dilated sufficiently [14, 23, 24] and a newly designed 0.025-inch guide wire was utilized in some studies [25], we used a 19-gauge FNA needle in all cases, as it allowed the passage of a rapid infusion of contrast. More importantly, we used the thicker 0.035-inch guide wire during the procedure with no guide-wire damage experienced. The hardness of the 0.035-inch guide wire makes it easier for the retrieval balloon to advance forward and push the stone. This method also omits the step of replacing the guide wire, which was reported in previous literature [15].

We directly used the co-axial cystotome to dilate the fistula, as it is fast and efficient, as recommended in one piece of literature [23]. A stiff catheter can also be used gently to dilate the tract [23] but a subcapsular hepatic hematoma may appear due to difficulty in advancing the transhepatic dilation catheter [12]. In addition, we usually used balloon dilators with markers in order to ensure the location under fluoroscopy. We dilated the biliary opening by using a balloon that was slightly larger than the largest stone seen on the cholangiogram, but it should not exceed the diameter of the bile duct.

There is a risk of complications during the procedure, such as bile leakage, perforation, post-procedure pancreatitis, and hemorrhage. Adverse events happened in two of the patients (15.4%). In Case 4, the guide wire dropped out from the biliary system after stone extraction; we did not puncture again to place a stent or NB tube. Although one asymptomatic fluid collection event happened after the procedure, it resolved spontaneously without any treatment. In Case 8, bile leakage and bile peritonitis developed, maybe because we punctured the bile duct a second time due to guide-wire dislodgement after dilation of the tract. Theoretically, post-procedure pancreatitis may happen because we have to dilate the papilla, similarly to performing ERCP. Maybe due to our small study sample, none of our patients developed pancreatitis. Post-procedure hemorrhage did not happen, although there is a report about hepatic subcapsular hematoma [12]. The following two points are important to avoid hemorrhage: (1) we tried to avoid the blood vessels along the puncture path; (2) even if blood vessels are pierced, liver parenchyma would tamponade the biliary drainage tube or stent in the transhepatic tract and decrease the chance of hemorrhage.

Single-session TASR seemed to be relatively safe and feasible for patients with SAA. Here, we have demonstrated that simplified single-session TASR has a high success rate and low incidence of adverse events, and shared the simplified operation process and experience. But it is still challenging technology for now due to a high degree of operation difficulty and the lack of dedicated devices, and further studies are needed to determine this procedure.

## Supplementary Material

goae056_Supplementary_Data
