# Supplementary material for: Simplified single-session EUS-guided transhepatic antegrade stone removal for management of choledocholithiasis in patients with surgically altered anatomy
Source: Gastroenterol Rep (Oxf). 2024 Jun 25;12:goae056. doi: 10.1093/gastro/goae056 (PMC11199342; doi:10.1093/gastro/goae056)
Supplement: goae056_Supplementary_Data [file goae056_supplementary_data.docx]

Supplementary Table 1.Details in TASR

| Patient number | Type of SAA | Operation time (min) | Bile duct width (mm) | Distance betweenbile duct and puncture site(mm) | Largest Size of stone (cm) | Diameter of dilation balloon(mm) | Drainage method | Adverse events |
| --- | --- | --- | --- | --- | --- | --- | --- | --- |
| 1 | Hepaticojejunostomy with Roux-en-Y anastomosis | 52 | 6 | 33.4 | 0.9*1.0 | 10mm | NB tube | None |
| 2 | Whipple | 80 | 4 | 18 | 0.8*1.0 | 11mm | Stent | None |
| 3 | Gastrectomy with Roux-en-Y anastomosis | 60 | 5.2 | 24.9 | 1.2*0.7 | 12mm | Stent | None |
| 4 | Billroth II | 60 | 3.7 | 30.2 | 1.0*0.9 | 10mm | None | Peritoneal effusion |
| 5 | Hepaticojejunostomy with Roux-en-Y anastomosis | 77 | 2.4 | 23.7 | 1.1*1.1 | 12mm | Stent | None |
| 6 | Gastrectomy with Roux-en-Y anastomosis | 63 | 5.5 | 42.6 | 1.0*1.2 | 12mm | Stent | None |
| 7 | Gastrectomy with Roux-en-Y anastomosis | 40 | 4.8 | 27.7 | 1.2*1.3 | 15mm | Stent | None |
| 8 | Whipple | 110 | 6.5 | 37 | 1.0*1.2 | None | Stent | Bile leak and bile peritonitis |
| 9 | Whipple | 61 | 7.2 | 38.4 | 0.8*0.9 | 8mm | Stent | None |
| 10 | Hepaticojejunostomy with Roux-en-Y anastomosis | 59 | 4.6 | 30.1 | 1.5*1.6 | 15mm | Stent | None |
| 11 | Whipple | 58 | 5.1 | 23.4 | 1.0*1.5 | 12mm | Stent | None |
| 12 | Gastrectomy with Roux-en-Y anastomosis | 60 | 2.5 | 47.2 | 1.5*0.8 | 12mm | Stent | None |
| 13 | Hepaticojejunostomy with Roux-en-Y anastomosis | 57 | 5 | 15 | 1.5*8.9 | 12mm | Stent | None |

NB, Naso-biliary; SAA, surgically altered anatomy.
